# Change of Mandibular Position during Two-Phase Orthodontic Treatment of Skeletal Class II in the Chinese Population

**DOI:** 10.1155/2015/804831

**Published:** 2015-01-28

**Authors:** Rhonda Nga Yi Cheung, Urban Hägg, Ricky Wing Kit Wong, Chongshan Liao, Yanqi Yang

**Affiliations:** ^1^Orthodontics, Faculty of Dentistry, The University of Hong Kong, 34 Hospital Road, Sai Ying Pun, Hong Kong; ^2^Department of Dentistry & Maxillofacial, United Christian Hospital, Kowloon, Hong Kong

## Abstract

The aim of this study was to evaluate the change in mandibular position during a two-phase orthodontic treatment of skeletal Class II malocclusion. Thirty consecutively treated Chinese male adolescents who had undergone two-phase treatment with Herbst appliance and fixed appliance and fulfilled the specific selection criteria were sampled. Cephalograms taken at T0 (before treatment), T1 (at the end of functional appliance treatment), and T2 (at the end of fixed appliance treatment) were analyzed. The change in sagittal positioning of the mandible was 6.8±3.44 mm in phase I (T0-T1), 0.4±2.79 mm in phase II (T1-T2), and 7.2±4.61 mm in total. The mandible came forward in 100% of the patients at T1. In phase II, it came forward in one-third (positive group) remained unchanged in one-third (stable group) and went backward in one-third (negative group) of the patients. At T2, it came forward twice as much in the positive group compared to the negative group. Mandibular length was significantly increased in 100% of the patients in both phases. In conclusion, during the treatment with functional appliance, the mandibular prognathism increases in all patients, whereas during the treatment with fixed appliance there is no significant change in mandibular prognathism.

## 1. Introduction

Ever since the introduction of functional appliances more than a century ago, their potential effects on modifying growth have been a matter of controversy. Based on prospective clinical trials some investigators claimed that they have demonstrated that functional appliances modify growth [1–5], whereas others meant that the effects were limited to the dentoalveolar process only [6–8]. However, the recent randomized clinical trials (RCTs) on treatment of Class II malocclusion seem to agree on that functional appliances do not influence the Class II pattern to a clinical significant degree [9], that is, marked forward positioning of the mandible. Moreover, when there eventually was an effect it was short-lived [10]. However, many studies reported that the mandible increased in length due to functional appliance treatment, and since the mandible was not markedly positioned forward, the likely effect was the increase of the lower face height [11].

The concept of RCT is considered the golden standard in the hierarchy of evidence [12] and has been used to evaluate the effect of functional appliances, such as one-phase versus two-phase treatment of Class II malocclusion [13–17]. However, others claimed that RCTs are not suitable in the orthodontic context [18]. For instance, are the cephalometric measurements chosen to evaluate the treatment changes and effects reliable, valid, and accurate? How are the many and various factors, such as research design, sampling methods, inclusion criteria, treatment and observation periods, methods used for evaluation, patient compliance, timing of the pubertal growth, operator's experience, and the choice of appliance, taken into account? The inherit complexity of the nature of the problems investigated with simple formulas in those RCT studies makes direct comparison of the results impossible. Subsequently, the obvious limitations of the recent RCTs on Class II treatment seem to provide inconclusive results again.

Most of the previous studies on functional appliances have focused on single-phase treatment only [2, 19–25], despite, in the clinical situation, second phase of fixed appliance therapy is often necessary in order to obtain a proper alignment and occlusion of the dentition. However, few studies had been carried out on two-phase treatment [13, 26, 27], and the effects of fixed appliance on the skeletal and dental changes after the first phase functional appliance treatment have not been fully investigated. Therefore, the purpose of this study is to investigate the change in the mandibular position during each phase of a two-phase orthodontic treatment of skeletal Class II malocclusion.

## 2. Materials and Methods

### 2.1. Sample Selection

The sample was selected from an original group of 194 consecutively treated Chinese male patients who underwent Herbst appliance therapy at the Prince Philip Dental Hospital, Faculty of Dentistry, University of Hong Kong, from 1999 to 2010. In order to obtain a more homogeneous study sample, subjects were included only when the following criteria were fulfilled: at pretreatment (T0) (1) male aged between 11 and 16, (2) Wits appraisal with value greater than −1.5 (norm for Chinese: −4.5, SD 3.0) [28], (3) angle Class II malocclusion (at least half-unit Class II molar relationship), (4) a convex facial profile, and (5) no permanent teeth extracted. Other criteria were as follows: (6) participants had undergone two-phase treatment with cast-type Herbst appliance, followed by preadjusted edgewise appliance, (7) the retention period between phase I and phase II did not exceed 3 months, (8) phase I should not be less than 9 months, but not more than 20 months, (9) phase II should not exceed 40 months, and (10) lateral cephalograms were obtained in natural head posture, with the teeth in centric occlusion and the lips in relaxed position [29] at pretreatment (T0), immediate post-Herbst (T1), and post-edgewise treatment (T2).

A sample of 30 male patients was available for further analysis (power 80%; *α* = 0.05). The age at the start of Herbst appliance therapy was 13.4 ± 1.2 years, with a range from 11.3 years to 15.9 years (Table 1). The total observation period was longer than the actual treatment time, as the pretreatment lateral cephalograms were taken, on average, three months before treatment. There was also a retention period of not exceeding three months between phase I and phase II.

The study sample was subdivided into three groups for further analysis according to the difference in the change in mandibular prognathism during the second phase: those with an increase of OLp-Pg more than 1.0 mm become* the positive group;* change of OLp-Pg within 1.0 mm, the* stable group*; and with a decrease of OLp-Pg more than 1.0 mm, the* negative group*.

### 2.2. Cephalometric Analysis

For calibration, 15 lateral cephalograms of 5 subjects were hand-traced initially by one investigator (RNYC) and verified by another investigator (UH). All lateral cephalograms were manually-traced by the same investigator (RNYC) twice, with one-week interval, and the two sets of data were then averaged in order to reduce the measurement error in landmark identification [30]. All the hand-tracings were then digitized and measured by the CASSOS software (CASSOS Clinical Evaluation Version 2004, Soft Enable Technology Limited, China). No corrections were made for linear radiographic enlargement (approximately 7% in the median plane) [31].

**Figure 1:**
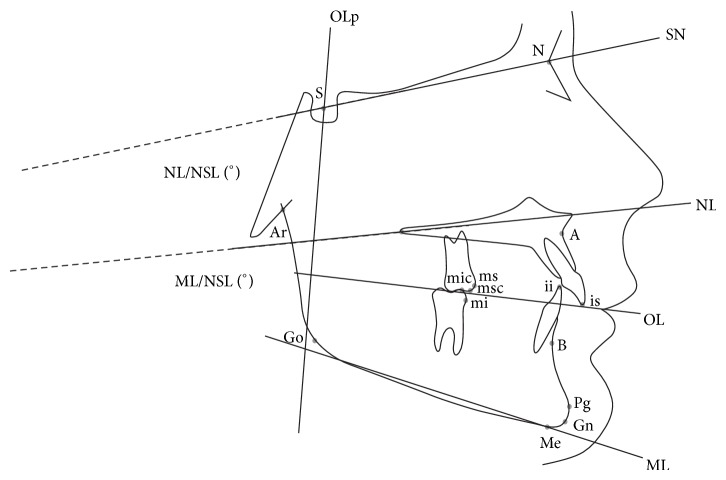
Cephalometric landmarks (for details please see [2, 19]). Cephalometric reference lines are OL: maxillary occlusal plane, the line joining the distobuccal cusp tip of the maxillary permanent first molar and the upper incisor tip, is, and OLp: occlusal plane perpendicular to OL through S. Sagittal variables: overjet (mm): is-OLp minus ii-OLp; mandibular length (mm): Ar-Gn; maxillary prognathism: A-OLp (mm) and S-N-A (°); mandibular prognathism: Pg-OLp (mm) and S-N-B (°); jaw base relationship: A-Pg (mm), A-N-B (°), and A, B on functional occlusal plane (mm); upper and lower incisor changes: is-A (mm), is-OLp minus A-OLp and ii-Pg (mm), ii-OLp minus Pg-OLp; upper and lower first molar changes: ms-A (mm), ms-OLp minus A-OLp and mi-Pg (mm), mi-OLp minus Pg-OLp; and molar relationship: ms-mi (mm), ms-OLp minus mi-OLp. Vertical variables: overbite (mm): ii-OL; upper face height (mm): N-NL; lower face height (mm): NL-Me; total face height (mm): NSL-Me; upper and lower incisor changes (mm): is-NL and ii-ML; upper and lower first molar changes (mm): msc-NL and mic-ML. Rotational changes (°): NSL/NL and NSL/ML.

The analysis of skeletal and dental changes was performed according to the method modified from Pancherz [2, 19]. Template obtained from the first lateral cephalogram (T0) was superimposed on the subsequent lateral cephalograms using the structures of the anterior cranial base [32, 33] rather than on the nasion-sella line, as described originally by Pancherz [2, 19]. Other parameters were also included to facilitate comparison with other studies: Wits appraisal, S-N-A angle, S-N-B angle, A-N-B angle, the mandibular length (articulare to gnathion), the upper face height, lower face height, and the total face height (Figure 1).

### 2.3. Method Error Study

Prior to analysis, the treatment changes for 10 patients were assessed twice with a two-week interval to determine the method error. The magnitude of the combined method error (ME) in locating, superimposing, and measuring the changes of the different cephalometric landmarks was calculated by the Dahlberg's formula [34]: $\text{ME}=\sqrt{\sum d^{2}/2n}$, where *d* was the difference between the two measurements of a pair and *n* was the number of double measurements. Paired *t*-test was also performed to assess systematic error. The combined error was not statistically significant and did not exceed ±0.4 (mm or degree) for any of the variables investigated.

### 2.4. Statistical Analysis

Data analysis was performed with statistical analysis computer software (IBM SPSS Statistics 19.0.0, IBM Corporation, Route 100, Somers, NY 10589, US). The normality (Kolmogorov-Smirnov test) of the data appeared to be valid. The arithmetic mean (mean) and standard deviation (SD) for each variable were calculated. Independent *t*-test was carried out to compare the differences between groups. Paired *t*-test with Bonferroni correction and one-way ANOVA were used to examine the difference between the changes observed. Significance was set at *α* = 0.05.

## 3. Results

### 3.1. Dentofacial Morphology of the Study Group (Table 2(a)) and the Subgroups (Table 2(b))

There were statistically significant differences in the dentofacial morphology between the study sample and the population norms [28, 35]. The study sample presented with Class II jaw base relationships as indicated by the Wits appraisal, significant increase in A-Pg and A-N-B angle; Class II molar relationship as indicated by the significant increase in ms-mi; significantly larger overjet, more prognathic maxilla in terms of OLp-A and more retrusive mandible in terms of S-N-B angle; increased upper face height; overerupted lower incisors relative to the mandibular plane; and overerupted maxillary molars relative to the maxillary plane.

When compared with the population norm, the positive group has a more prognathic maxilla (OLp-A) and normal mandible (OLp-Pg); the stable group has a relatively normal maxilla but more retrusive mandible, while the negative group has a more prognathic maxilla and more retrognathic mandible. The positive group has significant increase in overbite and decrease in mandibular plane angle (ML/NSL) when compared with the stable and negative groups and also the population norms, while the negative group has a significant increase in the upper face height (N-NL) when compared with the other two subgroups and the population norms.

### 3.2. Treatment Changes during the First Phase (T0-T1) Therapy with Cast-Herbst Appliance in the Study Group (Table 3(a)) and the Subgroups (Table 3(b))

**Figure 2:**
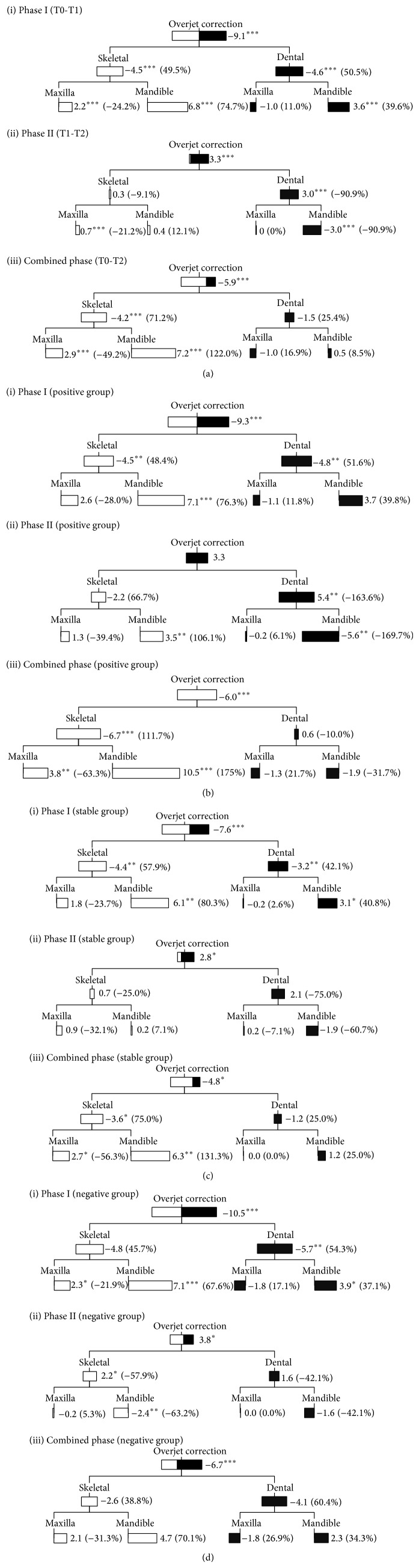
Overjet correction of (a) the whole study sample, (b) positive group, (c) stable group, and (d) negative group for phase I (T0-T1), phase II (T1-T2), and the total treatment period (T0–T2) (^*^ *P* < 0.05; ^**^ *P* < 0.01; ^***^ *P* < 0.001).

**Figure 3:**
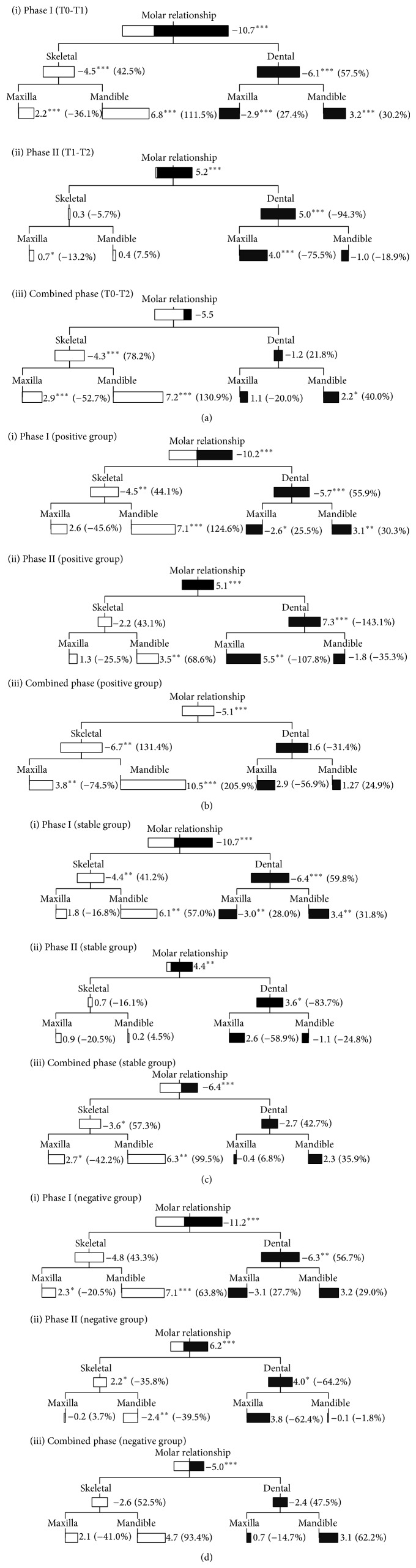
Molar correction of (a) the whole study sample, (b) positive growth group, (c) stable growth group, and (d) negative growth group for phase I (T0-T1), phase II (T1-T2), and the total treatment period (T0–T2) in the positive growth group (^*^ *P* < 0.05; ^**^ *P* < 0.01; ^***^ *P* < 0.001).

The statistically significant treatment changes during phase I were the decrease in overjet and overbite, increased maxillary prognathism (OLp-A), mandibular prognathism (OLp-Pg and S-N-B angle), mandibular growth (Ar-Gn) and upper and lower face heights (N-NL and NSL-Me), improvement in jaw base (Wits appraisal, A-Pg, and A-N-B angle) and molar relationships, protrusion of lower incisors, and eruption of lower molars. About 50% of overjet correction was due to the change in skeletal base (Figure 2(a)); skeletal effect contributed less (42.5%) than dentoalveolar effect (57.5%) in the correction of molar relationship (Figure 3(a)). No significant differences were observed among the three subgroups during the first phase treatment.

### 3.3. Treatment Changes during the Second Phase (T1-T2) Therapy with Preadjusted Edgewise Appliance in the Study Group (Table 3(a)) and the Subgroups (Table 3(b))

During phase II, the statistically significant effects observed were rebound in overjet and overbite, further increase in maxillary prognathism and mandibular growth, retroclination of lower incisors, mesialization of maxillary molars, deterioration of the molar relationship, further increase in the upper and lower face heights, and eruption of upper and lower incisors and molars (Figures 2(a) and 3(a)). During this phase, one-third of the patients continued to have forward positioning of the mandible (positive group) (Figures 2(b) and 3(b)). One-third remained relatively unchanged (stable group) (Figures 2(c) and 3(c)), while the remaining one-third showed a decrease in mandibular prognathism (negative group) (Figures 2(d) and 3(d)). Apart from forward positioning of the mandible, the positive group has a more significant increase in mandibular length, improvement in jaw base relationship, more retroclined lower incisors, and smaller mandibular plane angle when compared with the stable and negative groups.

### 3.4. Treatment Changes of the Overall Treatment Period (T0–T2) in the Study Sample (Table 3(a)) and the Subgroups (Table 3(b))

The statistically significant treatment changes observed for the combined phases were improvement in overjet and overbite, increase in maxillary and mandibular prognathism, increase in upper and lower face heights, enhanced mandibular growth, improvement in jaw base relationship, mesialization of mandibular molars, and eruption of upper and lower molars. For the total treatment period, 71.2% of overjet reduction was contributed by skeletal effect; dentoalveolar changes played a less significant role (Figure 2(a)). The same is also true for the improvement in molar relationship. Skeletal change contributed 78.2%, while dentoalveolar effects only contributed 21.8% in its overall correction (Figure 3(a)).

For the positive group, improvements in overjet and molar relationship were completely due to skeletal changes (Figures 2(b) and 3(b)). Skeletal effects contributed about 75% to overjet reduction in the stable group; only 25% was due to dentoalveolar effects (Figure 2(c)). For the correction of molar relationship, about 57% was due to skeletal effects, 43% was contributed by dentoalveolar changes (Figure 3(c)). For the negative group, dentoalveolar effects were more dominant in the overjet correction (about 40% skeletal and 60% dental) (Figure 2(d)), while skeletal and dentoalveolar effects played equal parts in the improvement in the molar relationship (Figure 3(d)).

## 4. Discussion

In this retrospective clinical study, strict inclusion criteria were adopted in order to obtain a more homogenous study sample that presented with skeletal Class II for evaluation. Linear cephalometric measurements are known to be more accurate and reliable than angular measurements [36, 37]. Angular measurements, such as A-N-B angle, are not valid for assessment of changes in the jaw base position during growth because reduction in A-N-B angle could have occurred, for instance, as a result of differential growth between the nasion and the A-point. Such change might exaggerate the restraint of maxillary prognathism and mask the increase of mandibular prognathism. Therefore, Wits appraisal, together with the molar relationship, was used to identify patients with skeletal Class II instead. For evaluation of treatment changes, a method modified from Pancherz [2, 19] was used. Increased overjet was not used to identify skeletal Class II because large overjet more than one or even two standard deviations (SD) to the population norms did not necessarily mean that the subjects have Class II jaw base relationship [38]. In fact, in one RCT study that used increased overjet as an inclusion criterion [13], the A-N-B angle of the sample ranged from 0.4 (−1 SD) to 12.2 (+4 SD) degrees, indicating that the sample was comprised of malocclusions ranging from mild skeletal Class III to very severe skeletal Class II.

Cast-Herbst appliance was chosen in the present study since it is particularly advantageous over other removable appliances as it works round the clock, and patient compliance is not an issue. Operators had experience handling the appliance, and laboratory technicians had fabricated the appliance for many years. Less breakage was observed than the banded type of Herbst. Even though more debond has been encountered with the cast-Herbst, the management is much easier [39].

Usually patients treated with Herbst appliance are more dentally mature, as anchorage requires fully erupted first premolars [40]. It was also recommended that, to take advantage of the increase in condylar growth and to reduce posttreatment retention time, Herbst therapy should be carried out close to the pubertal maximum of growth [41] to enhance the skeletal effects. There is only a small sex difference in the eruption of the first premolars, whereas the difference in the occurrence of the pubertal growth spurt differs 2 years [42]. In most RCT studies, data for the males and females were pooled despite the sex difference in the timing of puberty and the magnitude of growth. It was recommended that sexes should be analyzed separately especially in adolescence [41]. Therefore, only male patients who aged between 11 and 16 were included in this study, since they will not have passed their pubertal maximum before their first premolars have erupted and functional appliance therapy started, whereas many females might have passed their pubertal maximum before the specific anchorage teeth have erupted [42].

It was recommended by Herbst [43] that treatment duration with his appliance should not be less than 9 months, and an experimental study also suggested that sufficient time after forward positioning with a fixed jumping device was necessary to allow the newly formed condylar bone to mature and become stable, thus enabling normal growth to be maintained afterwards [44].

The study sample had Class II jaw base and molar relationships. They have significantly more prognathic maxilla and more retrusive mandible when compared with the population norms [28, 35]. This is in general agreement with another study on the dentofacial morphology on Chinese subjects with Class II malocclusion [45].

Significant improvement in overjet and overbite was observed after Herbst appliance therapy in 100% of the study sample, with protrusion of lower incisor and eruption of lower molars. Normalization of the jaw base and molar relationships was accomplished by significant forward positioning of the mandible and increase in mandibular length, distalization of upper molars, and mesialization of lower molars. The average increase in forward positioning of the mandible was 6.8 mm in 13 months, which is more than twice of that of normal growth [46] and indicated that there is an enhancement effect on mandibular prognathism with the Herbst appliance. The changes observed were in general agreement with the previous studies [3, 5, 25, 27, 40]. However, significant increase in the upper and lower face heights was also observed, which is in contrast with some of the studies [21, 27, 47].

After the Herbst appliance treatment in phase I, mandibular prognathism increased in 100% of the patients, but during phase II, only one-third of the patients continued to have forward positioning of the mandible. In one-third of the patients the mandible remained relatively unchanged, while in the remaining one-third the mandibular prognathism decreased (Figure 3(a)). However, significant increase in mandibular length was still observed in 100% of the patients in phase II. Such findings indicated that, instead of “subnormal” growth in the post-Herbst period, the mandible continued to grow in the stable and negative groups, but mainly vertically rather than sagittally.

For the positive group, the overall (T0–T2) improvements in overjet and molar relationship were completely due to skeletal changes (Figures 2(b) and 3(b)). In the negative group dentoalveolar effects were more dominant in overjet correction (Figure 2(d)), while skeletal and dentoalveolar effects played equal parts in the normalization of molar relationship (Figure 3(d)). Moreover, during phase II, the lower incisors were significantly more retroclined in the positive group compared with the other two groups. Therefore, a good vertical control of the maxilla and sagittal control of the incisor inclination may be beneficial to the correction of skeletal Class II malocclusion.

For the total treatment period (T0–T2), there were significant improvement in the study sample in overjet and overbite, with normalization of the jaw base and molar relationships, and increase in upper and lower face heights. During phase I, skeletal changes contributed 49.5% and 42.5% to the overjet and molar corrections, respectively, whereas at the end of phase II, due to dentoalveolar relapse, contributions from skeletal changes increased to 71.2% and 78.2%, respectively (Figures 2(a) and 3(a)). Such findings were in conflict with some of the previous studies, which concluded that functional appliance treatment does not have beneficial clinical effect in the correction of skeletal Class II malocclusion [11, 13, 48]. The differences in treatment changes in the three subgroups demonstrated clearly the large variation in treatment response with the same orthodontic appliance, which might reflect that the underlying growth pattern differs between groups, and also reflect the weakness of current diagnostic methods. However, as stated before, due to the shortcomings of RCTs in evaluating the orthodontic treatment outcomes, conclusions valid in the clinical context still could not be drawn.

The limitations of the present study are the small sample size and no comparable control group. The small sample size obtained under the specific inclusion criteria did not allow us to investigate if there were differences in the treatment changes between patients who were treated with headgear-Herbst or Herbst only, maximum or stepwise advancement of the bite during the Herbst treatment, or extraction and nonextraction therapy. However, these different modes of treatment were found in all three subcategories, so there was no obvious bias in this context.

Ideally, it had been desirable with a matched control group, but it is neither practical nor ethical to leave a group of growing patients seeking treatment untreated for a longer period of time. In fact none of the RCTs has a control group for the whole two-phase treatment. If they had a control group, it was for the first phase only [14].

Future studies have to be planned with great skills and caution, in attempt to minimize the unavoidable flaws. Meanwhile, those colleagues who use functional appliances on selected cases and in a skilled manner seem to have no sound scientific reason not to continue to do so.

## 5. Conclusions

1. During phases I and II, the mandible increases in length.
2. During phase I with Herbst appliance, there is an increase in mandibular prognathism.
3. During phase II with fixed appliance, the direction of growth of the mandible varies.

## Figures and Tables

**Table 1:** Age at the beginning of phase 1 (T0), end of phase 1/beginning of phase 2 (T1) and end of treatment (T2), and duration of the treatment phases of the study sample (*N* = 30) and the three subgroups (*n* = 10 in each subgroup).

	Average age (years )			Average time of treatment phases (years)		
	T0	T1	T2	T0-T1	T1-T2	T0–T2
Whole study sample	13.4 ± 1.20	14.5 ± 1.20	16.4 ± 1.50	1.1 ± 0.20	1.9 ± 0.80	3.0 ± 0.90
Positive group	12.8 ± 1.07^ns^	13.9 ± 1.12^ns^	16.2 ± 1.92^ns^	1.1 ± 0.27^ns^	2.2 ± 0.74^ns^	3.3 ± 0.82^ns^
Stable group	13.9 ± 1.21^ns^	15.0 ± 1.17^ns^	16.6 ± 1.37^ns^	1.1 ± 0.26^ns^	1.6 ± 0.75^ns^	2.7 ± 0.87^ns^
Negative group	13.4 ± 1.07^ns^	14.5 ± 1.08^ns^	16.4 ± 1.32^ns^	1.1 ± 0.25^ns^	1.8 ± 0.77^ns^	3.0 ± 0.85^ns^

SD: Standard deviation.

^ns^Non-significant, ^*^ *P* < 0.05, ^**^ *P* < 0.01, ^***^ *P* < 0.001.

**(a):** 

Variables	Population norm		T0		T1		T2	
	Mean	SD	Mean	SD	Mean	SD	Mean	SD
Sagittal	****							
Overjet (mm)	4.3	1.76	*9.8* ^***^	3.13	*0.6* ^***^	2.91	3.9	0.88
Wits appraisal (mm)	−4.5	3.00	*3.6* ^***^	3.58	−4.3	3.34	*0.2* ^***^	3.06
Maxillary prognathism								
OLp-A (mm)	76.1	3.86	*80.2* ^**^	4.25	*82.4* ^***^	4.22	*83.1* ^***^	4.25
S-N-A (°)	82.0	3.50	82.6	3.69	83.1	3.36	82.4	3.12
Mandibular prognathism								
OLp-Pg (mm)	82.3	4.88	*79.2* ^**^	6.74	*86.0* ^***^	8.30	*86.4* ^***^	8.99
OLp-B (mm)	N/A	N/A	77.0	5.93	83.6	7.34	83.4	7.66
Ar-Gn	N/A	N/A	104.0	5.78	112.0	6.88	113.9	7.73
S-N-B (°)	79.0	3.00	*75.1* ^***^	3.45	*77.7* ^*^	3.93	*77.1* ^**^	4.31
Jaw base relationship								
A-Pg (mm)	−6.2	3.38	*1.1* ^***^	3.76	*−3.5* ^***^	5.53	*−3.3* ^***^	5.99
A-N-B (°)	3.0	2.00	*7.5* ^***^	1.78	*5.4* ^***^	1.93	*5.3* ^***^	2.20
Maxillary incisor								
OLp-is (mm)	88.1	4.54	91.8	4.89	*93.0* ^***^	5.30	*93.7* ^***^	5.40
Mandibular incisor								
OLp-ii (mm)	83.8	4.44	82.0	4.75	*92.4* ^***^	5.79	*89.7* ^***^	5.13
Maxillary molar								
OLp-ms (mm)	55.0	3.98	56.7	4.30	56.0	5.07	*60.6* ^***^	5.30
Mandibular molar								
OLp-mi (mm)	57.7	4.26	54.6	4.95	*64.6* ^***^	6.02	*64.0* ^***^	5.88
Molar relationship								
ms-mi (mm)	−2.7	1.62	*2.1* ^***^	1.77	*−8.6* ^***^	2.33	*−3.4* ^*^	2.04
Vertical								
Overbite (mm)	2.2	1.51	3.2	2.60	*−0.1* ^***^	1.44	1.0	0.89
Upper face height								
N-NL (mm)	54.0	3.50	*57.2* ^***^	3.48	*59.7* ^***^	3.89	*60.7* ^***^	3.55
Lower face height								
NL-Me (mm)	64.0	4.00	65.5	4.54	*71.0* ^***^	5.34	*72.7* ^***^	5.52
Total face height								
NSL-Me (mm)	N/A	N/A	117.8	6.50	126.4	7.23	129.0	7.16
Incisor position								
is-NL (mm)	28.9	2.72	30.5	2.82	*32.9* ^***^	2.98	*33.7* ^***^	3.14
ii-ML (mm)	41.3	2.73	*43.9* ^**^	3.35	*43.4* ^**^	3.68	*44.8* ^***^	3.72
Molar position								
msc-NL (mm)	21.9	2.22	*23.7* ^*^	2.25	23.1	2.74	*26.0* ^***^	3.03
mic-ML (mm)	31.8	2.41	33.2	2.25	*35.9* ^***^	2.54	*37.8* ^***^	3.09
Rotational changes								
NL/NSL (°)	9.1	3.34	9.8	2.46	9.8	3.00	9.4	3.26
ML/NSL (°)	35.3	5.57	34.8	7.28	34.0	7.49	33.6	8.38

SD: Standard deviation.

^*^ *P* < 0.05, ^**^ *P* < 0.01, ^***^ *P* < 0.001.

**(b):** 

Variables	Population norm		Positive group		Stable group		Negative group	
	Mean	SD	Mean	SD	Mean	SD	Mean	SD
Sagittal								
Overjet (mm)	4.3	1.76	*10.3* ^***^	2.54	*8.6* ^***^	3.45	*10.3* ^***^	3.34
Wits appraisal (mm)	−4.5	3.00	*3.7* ^***^	2.60	*3.4* ^***^	2.85	*3.7* ^***^	5.13
Maxillary prognathism								
OLp-A (mm)	76.1	3.86	*80.6* ^***^	3.90	78.2	4.30	*81.9* ^***^	4.04
SNA (°)	82.0	3.50	83.3	3.87	81.4	3.45	83.0	3.83
Mandibular prognathism								
OLp-Pg (mm)	82.3	4.88	81.0	5.65	*77.5* ^**^	8.04	*79.0* ^*^	6.54
OLp-B (mm)	N/A	N/A	78.2	5.54	75.3	7.10	77.5	5.21
Ar-Gn	N/A	N/A	104.9	6.08	101.8	5.07	105.4	6.05
SNB (°)	79.0	3.00	*76.4* ^***^	4.26	*74.2* ^***^	3.07	*74.7* ^***^	2.83
Jaw base relationship								
A-Pg (mm)	−6.2	3.38	*−0.4* ^***^	2.98	*0.7* ^***^	4.40	*2.9* ^***^	3.35
ANB (°)	3.0	2.00	*6.9* ^***^	2.03	*7.2* ^***^	1.50	*8.3* ^***^	1.65
Maxillary incisor								
OLp-is (mm)	88.1	4.54	*92.6* ^***^	4.64	88.6	3.64	*94.2* ^***^	4.89
Mandibular incisor								
OLp-ii (mm)	83.8	4.44	82.3	4.59	80.0	4.51	83.9	4.80
Maxillary molar								
OLp-ms (mm)	55.0	3.98	56.4	4.53	56.0	4.68	57.7	3.93
Mandibular molar								
OLp-mi (mm)	57.7	4.26	54.2	5.01	53.6	5.44	56.1	4.51
Molar relationship (mm)								
ms-mi (mm)	−2.7	1.62	*2.1* ^***^	1.81	*2.4* ^***^	1.70	*1.6* ^***^	1.88
Vertical								
Overbite (mm)	2.2	1.51	*4.4* ^***^	1.61	2.9	2.93	2.2	2.76
Upper face height								
N-NL (mm)	54.0	3.50	56.4	2.68	56.0	2.27	*59.2* ^***^	4.48
Lower face height								
NL-Me (mm)	64.0	4.00	64.3	4.07	64.1	2.70	68.0	5.63
Total face height								
NSL-Me (mm)	N/A	N/A	116.6	5.17	114.7	4.52	122.2	7.40
Incisor position								
is-NL (mm)	28.9	2.72	30.3	2.84	30.0	2.48	*31.1* ^*^	3.27
ii-ML (mm)	41.3	2.73	*43.5* ^*^	2.21	42.3	1.72	*45.9* ^***^	4.56
Molar position								
msc-NL (mm)	21.9	2.22	*23.5* ^*^	2.38	*23.6* ^*^	1.96	*23.9* ^**^	2.59
mic-ML (mm)	31.8	2.41	32.8	2.22	32.8	1.98	*34.1* ^**^	2.51
Rotational changes								
NL/NSL (°)	9.1	3.34	8.8	2.04	*11.3* ^*^	2.46	9.4	2.33
ML/NSL (°)	35.3	5.57	*31.1* ^*^	8.11	35.1	6.40	38.1	6.00

SD: Standard deviation.

^*^ *P* < 0.05, ^**^ *P* < 0.01, ^***^ *P* < 0.001.

**(a):** 

Variables	T0-T1		T1-T2		T0–T2	
	Mean	SD	Mean	SD	Mean	SD
Sagittal						
Overjet (mm)	*−9.1* ^***^	3.96	*3.3* ^***^	3.00	*−5.9* ^***^	3.10
Wits appraisal (mm)	*−7.9* ^***^	3.51	*4.5* ^***^	2.93	*−3.4* ^***^	3.57
Maxillary prognathism						
OLp-A (mm)	*2.2* ^***^	1.94	*0.7* ^*^	1.60	*2.9* ^***^	2.08
SNA (°)	0.5	1.94	*−*0.6	2.00	*−*0.1	2.27
Mandibular prognathism						
OLp-Pg (mm)	*6.8* ^***^	3.44	0.4	2.79	*7.2* ^***^	4.67
OLp-B (mm)	*6.6* ^***^	3.18	*−*0.2	2.59	*6.4* ^***^	4.22
Ar-Gn	*8.0* ^***^	2.71	*1.9* ^***^	1.66	*9.9* ^***^	3.83
SNB (°)	*2.6* ^***^	1.57	*−*0.6	1.66	*2.0* ^***^	2.38
Jaw base relationship						
A-Pg (mm)	*−4.5* ^***^	3.03	0.3	2.51	*−4.2* ^***^	3.57
ANB (°)	*−2.1* ^***^	1.66	0.0	1.47	*−2.1* ^***^	1.73
Maxillary incisor						
OLp-is-OLp-A (mm)	*−*1.0	3.97	0.0	2.51	*−*1.0	4.19
Mandibular incisor						
OLp-ii-OLp-Pg (mm)	*3.6* ^***^	2.85	*−3.0* ^***^	2.97	0.5	3.59
Maxillary molar						
OLp-ms-OLp-A (mm)	*−2.9* ^***^	2.04	*4.0* ^***^	3.41	1.1	3.20
Mandibular molar						
OLp-mi-OLp-Pg (mm)	*3.2* ^***^	1.93	*−*1.0	2.71	*2.2* ^*^	3.35
Molar relationship						
ms-mi (mm)	*−10.7* ^***^	2.45	*5.2* ^***^	2.13	*−5.5* ^***^	2.43
Vertical						
Overbite (mm)	*−3.3* ^***^	2.26	*1.1* ^***^	1.49	*−2.2* ^**^	2.64
Upper face height						
N-NL (mm)	*2.5* ^***^	0.51	*1.1* ^***^	1.59	*3.5* ^***^	2.60
Lower face height						
NL-Me (mm)	*5.6* ^***^	2.07	*1.6* ^***^	1.89	*7.2* ^***^	2.65
Total face height						
NSL-Me (mm)	*8.6* ^***^	2.59	*2.6* ^***^	2.40	*11.2* ^***^	3.94
Incisor position						
is-NL (mm)	*2.4* ^***^	2.20	*0.9* ^***^	0.91	*3.2* ^***^	2.37
ii-ML (mm)	*−*0.5	2.30	*1.4* ^***^	1.90	0.9	2.41
Molar position						
msc-NL (mm)	*−*0.6	1.60	*2.9* ^***^	1.68	*2.3* ^***^	1.97
mic-ML (mm)	*2.6* ^***^	1.27	*2.0* ^***^	1.43	*4.6* ^***^	1.85
Rotational changes						
NL/NSL (°)	0.0	2.31	*−*0.4	2.12	*−*0.4	2.89
ML/NSL (°)	*−*0.7	1.90	*−*0.4	2.05	*−*1.1	2.49

SD: standard deviation.

^*^ *P* < 0.05, ^**^ *P* < 0.01, ^***^ *P* < 0.001.

**(b):** 

	T0-T1						T1-T2						T0–T2					
Variables	Positive growth		Stable growth		Negative growth		Positive growth		Stable growth		Negative growth		Positive growth		Stable growth		Negative growth	
	Mean	SD	Mean	SD	Mean	SD	Mean	SD	Mean	SD	Mean	SD	Mean	SD	Mean	SD	Mean	SD
Sagittal																		
Overjet (mm)	−*9.3* ^***^	3.10	−*7.6* ^**^	4.10	−*10.5* ^***^	4.38	3.3	3.98	*2.8* ^*^	1.91	*3.8* ^*^	2.96	−*6.0* ^***^	2.51	−*4.8* ^*^	3.29	−*6.7* ^**^	3.44
Wits appraisal (mm)	−*7.6* ^***^	3.42	−*7.3* ^***^	2.61	−*8.7* ^**^	4.46	3.3	3.31	*4.5* ^**^	2.28	*5.6* ^**^	2.94	−*4.3* ^*^	3.23	−2.9	2.89	−3.2	4.58
Maxillary prognathism																		
OLp-A (mm)	2.6	2.21	1.8	1.99	*2.3* ^*^	1.72	1.3	1.22	0.9	1.84	−0.2	1.40	*3.8* ^**^	2.23	*2.7* ^*^	1.61	2.1	2.13
SNA (°)	0.6	2.29	0.3	2.23	0.6	1.39	−0.2	1.67	−0.4	1.84	−1.4	2.42	0.4	2.73	0.0	1.77	−0.7	2.31
Mandibular prognathism																		
OLp-Pg (mm)	*7.1* ^***^	3.08	*6.1* ^**^	3.06	*7.1* ^***^	4.31	*3.5* ^**^	2.04	0.2	0.67	−*2.4* ^**^	1.08	*10.5* ^***^	4.55	*6.3* ^**^	3.23	4.7	4.36
OLp-B (mm)	*7.0* ^***^	2.57	*5.8* ^**^	3.07	*7.0* ^**^	3.94	2.3	1.94	0.0	0.57	−*3.0* ^**^	1.54	*9.2* ^***^	4.11	*5.8* ^**^	3.19	4.1	3.89
Ar-Gn	*8.5* ^***^	3.53	*7.7* ^***^	2.29	*7.8* ^***^	2.32	*2.9* ^**^	1.69	*1.6* ^**^	0.86	1.1	1.81	*11.5* ^***^	4.88	*9.3* ^***^	2.69	*8.9* ^***^	3.45
SNB (°)	*2.8* ^**^	1.69	*2.2* ^**^	1.26	*2.8* ^*^	1.80	0.6	1.72	−0.5	0.92	−1.8^*^	1.37	3.4	2.78	*1.7* ^*^	1.06	1.0	2.46
Jaw base relationship																		
A-Pg (mm)	−*4.5* ^**^	2.73	−*4.4* ^**^	2.29	−4.8	4.13	−2.2	2.20	0.7	1.57	*2.2* ^*^	1.41	−*6.7* ^**^	3.22	−*3.6* ^*^	2.44	−2.6	3.69
ANB (°)	−*2.2* ^*^	1.70	−*1.9* ^*^	1.36	−2.2	1.99	−0.8	1.32	0.2	1.58	0.5	1.35	*−3.0* ^**^	1.43	−1.7	1.45	−1.8	2.05
Maxillary incisor																		
OLp-is-OLp-A (mm)	−1.1	2.27	−0.1	5.33	−1.8	3.95	−0.2	3.03	0.2	2.12	0.0	2.56	−1.3	4.69	0.0	4.67	−1.8	3.32
Mandibular incisor																		
OLp-ii-OLp-Pg (mm)	3.7	3.64	*3.1* ^*^	2.10	*3.9* ^*^	2.84	−*5.6* ^**^	3.22	−1.9	1.73	−1.6	2.00	−1.9	3.74	1.2	2.64	2.3	3.18
Maxillary molar																		
OLp-ms-OLp-A (mm)	−*2.6* ^*^	1.72	−*3.0* ^**^	1.69	−3.1	2.73	*5.5* ^**^	3.19	2.6	2.08	3.8	4.26	2.9	3.72	−0.4	2.09	0.7	2.93
Mandibular molar																		
OLp-mi-OLp-Pg (mm)	*3.1* ^**^	1.34	*3.4* ^**^	1.34	3.2	2.91	−1.8	3.28	−1.1	2.21	−0.1	2.52	1.3	4.12	2.3	2.06	3.1	3.60
Molar relationship																		
ms-mi (mm)	−*10.2* ^***^	1.70	−*10.7* ^***^	2.58	−*11.2* ^***^	3.03	*5.1* ^***^	1.86	*4.4* ^**^	1.14	*6.2* ^***^	2.84	−*5.1* ^***^	1.97	−*6.4* ^***^	2.49	−*5.0* ^***^	2.79
Vertical																		
Overbite (mm)	−*3.8* ^**^	2.33	−*3.4* ^*^	2.54	−*2.5* ^*^	1.88	*0.8* ^*^	1.84	*1.3* ^***^	0.58	1.2	1.82	−*3.1* ^*^	2.04	−2.2	2.92	−1.3	2.84
Upper face height																		
N-NL (mm)	*2.9* ^**^	1.85	2.6	2.46	1.9	1.65	0.9	1.91	1.1	1.51	1.2	1.48	3.8	3.24	*3.8* ^**^	2.20	*3.1* ^*^	2.48
Lower face height																		
NL-Me (mm)	*6.0* ^***^	2.47	*5.3* ^***^	1.90	*5.3* ^***^	1.95	*2.2* ^***^	2.46	*1.2* ^*^	1.01	1.5	1.98	*8.2* ^***^	3.51	*6.5* ^***^	1.74	*6.9* ^***^	2.37
Total face height																		
NSL-Me (mm)	*9.3* ^***^	3.29	*8.6* ^***^	2.52	*7.9* ^***^	1.80	*3.6* ^***^	2.91	*2.2* ^*^	1.65	2.2	2.45	*12.9* ^***^	4.91	*10.8* ^***^	3.06	*10.0* ^***^	3.42
Incisor position																		
is-NL (mm)	2.0	2.23	2.2	2.45	*2.9* ^*^	2.02	0.8	0.75	0.5	1.08	*1.2* ^*^	0.85	2.8	2.75	*2.8* ^*^	2.07	*4.1* ^**^	2.21
ii-ML (mm)	−0.5	2.51	−0.6	1.46	−0.4	2.92	1.3	2.58	*1.5* ^*^	1.12	1.3	1.94	0.8	2.89	0.9	1.20	0.9	2.99
Molar position																		
msc-NL (mm)	0.2	1.47	−0.8	1.10	−1.2	1.94	*3.3* ^***^	1.48	*2.0* ^*^	1.38	*3.4* ^**^	1.96	*3.5* ^***^	1.49	*1.3* ^*^	1.03	2.2	2.53
mic-ML (mm)	*2.9* ^**^	1.79	*2.6* ^***^	0.57	*2.4* ^**^	1.25	*1.8* ^**^	1.52	*2.1* ^**^	1.11	*2.0* ^*^	1.73	*4.7* ^**^	2.36	*4.7* ^***^	1.20	*4.4* ^***^	1.98
Molar position																		
NL/NSL (°)	0.7	2.43	0.0	2.71	−0.9	1.63	−1.5	2.54	−0.2	1.73	0.6	1.63	−0.8	3.71	−0.2	2.68	−0.3	2.39
ML/NSL (°)	−0.3	1.79	−0.7	1.68	−1.2	2.26	−1.5	2.26	−0.4	1.45	0.8	1.86	−1.8	2.94	−1.1	2.06	−0.4	2.47

SD: standard deviation.

^*^ *P* < 0.05, ^**^ *P* < 0.01, ^***^ *P* < 0.001.
